# Prevalence of and Risk Factors for Asymptomatic Inﬂammatory (NIH-IV) Prostatitis in Chinese Men

**DOI:** 10.1371/journal.pone.0071298

**Published:** 2013-08-13

**Authors:** Chunlei Wu, Zhifu Zhang, Zheng Lu, Ming Liao, Youjie Zhang, Yuanliang Xie, Xuefeng Guo, Xiaoxiang Yu, Xiaobo Yang, Yong Gao, Aihua Tan, Zengnan Mo

**Affiliations:** 1 Institute of Urology and Nephrology, First Affiliated Hospital of Guangxi Medical University, Nanning, Guangxi Zhuang Autonomous Region, China; 2 Center for Genomic and Personalized Medicine, Guangxi Medical University, Nanning, Guangxi Zhuang Autonomous Region, China; 3 Department of Occupational Health and Environmental Health, School of Public Health of Guangxi Medical University, Nanning, Guangxi Zhuang Autonomous Region, China; 4 Department of Urology, The 303rd Hospital of Chinese People's Liberation Army, Nanning, Guangxi Zhuang Autonomous Region, China; Northwestern University, United States of America

## Abstract

**Background:**

While many investigators have studied symptomatic prostatitis, little research has been done with regard to asymptomatic (NIH-IV) prostatitis.

**Purpose:**

To describe the prevalence of and risk factors for NIH-IV prostatitis among a large male population.

**Methods:**

The study population was comprised of 1,868 men at the second phase recruitment of a population-based cohort in China. Asymptomatic and symptomatic men were defined by the National Institutes of Health Chronic Prostatitis (CP) Symptom Index. Meanwhile, EPS specimens and their leukocyte count were collected. Lifestyle and demographic characteristics were obtained through a questionnaire.

**Results:**

Prevalence of NIH-IV prostatitis was 21.1% among 1,868 asymptomatic men aged 19–78 years and increased with age. After adjusteing for potential confounding variables (age, smoking habits, alcohol drinking habits, education, physical activity, hypertension, dyslipidemia, obesity and diabetes), age remained a significant factor for NIH-IV prostatitis (OR = 1.35; 95% CI = 1.06–1.71; *P* = 0.01) and the risk of NIH-IV prostatitis was significantly higher in smokers≧15 pack/years than non-smokers (OR = 1.33; 95% CI = 1.01–1.75; *P* = 0.03). In addition, compared with non-drinkers, the OR of NIH-IV prostatitis in drinkers ≧1 drinks/week was 1.35 (95% CI = 1.03, 1.77, *p* = 0.02) after adjusting for the other variables above. In addition, having less than a college education may be a risk factor for NIH-IV prostatitis, although a statistically significant difference did not exist in our data (OR = 1.22; 95% CI = 0.97–1.52; *P* = 0.08).

**Conclusions:**

Our findings suggest that NIH-IV prostatitis is prevalent in China. Age, smoking, drinking and lower education levels were associated with an increased risk of NIH-IV prostatitis. The prevalence of NIH-IV prostatitis should be taken into account when estimating the total prevalence of CP in future studies.

## Introduction

Prostatitis is a common yet uncertain condition without clear diagnostic criteria and treatment strategies [1]. In 1995, The National Institutes of Health (NIH) organized the International Prostatitis Collaborative Network workshop and proposed a new classification of prostatitis, and since then asymptomatic inﬂammatory (NIH category IV) prostatitis has been regarded as a separate clinical entity among other prostatitis syndromes [2]. This condition is characterized by the presence of inﬂammatory cells in expressed prostatic secretion or in histologically prostate biopsy specimens in an otherwise asymptomatic patient and is therefore diagnosed solely in the laboratory.

Unfortunately, while many investigators have studied symptomatic prostatitis [3], [4], [5], [6], [7], little research has been done with regard to asymptomatic prostatitis. Although some studies have suggested a relationship between NIH-IV prostatitis and serum prostate specific antigen (PSA) levels [8], [9], [10], and others have tried to describe the prevalence of NIH-IV prostatitis [11], [12], little is known about the effects of factors such as social characteristics, lifestyle, hypertension, diabetes, dyslipidemia, obesity and diabetes on NIH-IV prostatitis. Therefore, we analysed information from the Fangchenggang Area Male Health and Examination Survey (FAMHES) to describe the prevalence of and risk factors for NIH-IV prostatitis among a Chinese male population.

## Materials and Methods

### Study Participants

The Fangchenggang Area Male Health Examination Survey (FAMHES) is a population-based epidemiological cohort study in the area of Guangxi, China, aiming at investigating the influence of environmental and genetic factors on the health of males and the progress of age-related chronic diseases, FAHMES is planning to make a follow up survey every two years afterthe initial two stages have been completed. In the first phase of the survey, a comprehensive demographic and health survey was conducted on 4,303 continuous males who voluntarily participated in the large-scale physical examination in Fangchenggang First People’s Hospital Medical Centre from September 2009 to December 2009. A detailed procedure of the first stage has been described elsewhere. [13]. Based on the first phase of the survey, the second phase (FAMHES II) follows up the survey by expanding the sample size from 4303 to 5988 subjects (3500 newly enrolled) from July 2011 to November 2011. A total of 5540 people completed the data collection interviews. The current study analysis is of the data from the second phase. All subjects provided written informed consent, and the study was approved by the Ethics and Human Subject Committee of Guangxi Medical University.

In the current cross-sectional study, participants were excluded based on the following criteria: (1) did not fill in the NIH Chronic Prostatitis Symptom Index (NIH-CPSI) questionnaire or offered incomplete individual information; (2) with any complaints of chronic pelvic pain or discomfort, abnormal mid void urine, or urinary tract infection; (3) refused a digital rectal examination or no expressed prostatic secretion (EPS) specimen could be obtained; (4) self-reported having specific (e.g. a history of sexually transmitted disease) or nonspecific inﬂammation (e.g. urinary tract infections), or has taken antibiotic medication within the previous month; (5) has taken medication that inﬂuenced the genitourinary system within the previous month, including diuretics,αblockers, or 5-α-reductase inhibitors; (6) had urinary tract surgeries or trauma history; (7) has been diagnosed with a nervous system disease or mental illness, or took antipsychotic and depression medications (e.g. sedatives, antidepressant drugs, etc.). In the end we enrolled 1,868 men in the present study.

### Data Collection

Data collection was conducted in Fangchenggang First People’s Hospital Medical examination Centre. During the physical examination, a face-to-face interview was conducted by trained physicians. Data on demographic characteristics (age, education, occupation, etc.), lifestyle characteristics (smoking, alcohol consumption, and physical activity), health status, and medical history were collected using a standardized questionnaire.

#### Definition of NIH-IV prostatitis

All study participants were required to complete NIH-CPSI independently with investigators’ (physician) guidance. The NIH-CPSI is a self-administered and validated instrument [14], which had been translated into Chinese. This questionnaire consists of 3 parts for a total of 9 questions [15]. The first part (questions 1–4) evaluates the site, frequency, and severity of pain; the second part (questions 5 and 6) inquires about problems with voiding symptoms; and the third part (questions 7–9) assesses quality of life. Asymptomatic men were defined if they answered negatively to having symptoms of urinary discomfort and/or pain based on NIH-CPSI questionnaire.

Each participant was required to undergo a digital rectal examination for collecting an EPS specimen. Polypropylene containers containing EPS were brought to the clinic laboratory immediately. Wet mounts were made and examined promptly with a high power microscope (400×) [16], [17]. At least 25 fields were examined and leukocytes were counted and averaged to determine the mean number per high-power microscope (HP). Leukocyte clumps in the latter part of the study were noted and counted as accurately as possible. To distinguish the degree of inflammation in the EPS, Leukocyte count results were divided into 5 categories: occasional or few (0–9 leukocytes/HP), 1+(10–20 leukocytes/HP), 2+(21–30 leukocytes/HP), 3+(31–40 leukocytes/HP), and 4+ (>40 leukocytes/HP).

Finally, participants were classified as having NIH-IV prostatitis if we observed 10 or more WBC/HPF in EPSs among asymptomatic men as based on previous data [11], [18], [19].

#### Demographic characteristics

Because it has been suggested that prostatitis is a younger man’s disease, we took age as a categorical variable and divided it into 2 groups: <40 years and ≥40 years. Educatiom level of the participants was categorized into two groups, college and above, or below college.

#### Self-reported lifestyle

Self-reported smoking history was obtained from the questionnaire. The questionnaires asked if the men have ever smoked at least once a day for more than 6 months in his lifetime. Those answering ‘‘yes’’ were asked to report the average number of cigarettes smoked per day and for how many years they had smoked that amount. In addition, pack-years were calculated by multiplying the number of packs smoked per day (1 pack = 20 cigarettes) by the number of years smoked to evaluate the severity of smoking. Pack-years were categorized by median as <15and ≧15 pack-years. Alcohol consumption was defined as having at least one alcoholic drink such as beer, wine, and hard liquor per week for six consecutive months. Physical activity or inactivity was assessed subjectively from the questionnaire. Men with regular physical activity of two or more hours per week were considered active.

#### Anthropometric measurements

Anthropometric measurements were performed by trained personnel using a standardized protocol. Body weight and height were measured without shoes to the nearest 0.1 kg and 0.1 cm, respectively. Body mass index (BMI) was calculated as the ratio between weight (kg) and the height squared (m^2^). Obesity was defined as having a BMI ≧28 kg/m^2^ higher than the normal range of Chinese adult men [20]. Blood pressure was measured by trained nurses with a mercury sphygmomanometer on the right arm of the participants in a comfortable sitting position after at least a 5-minute rest. The presence of hypertension was defined by contractive pressure ≧140 mmhg and/or diastolic pressure ≧90 mmhg and/or the use of anti-hypertensive medications.

#### Serum assay

Overnight fasting venous blood specimens were collected between 8∶00 and 11∶00 in the morning from all subjects for measurement of serum lipids and plasma glucose. Triglycerides, high-density lipoprotein (HDL), low-density lipoprotein (LDL), cholesterol and serum glucose were measured enzymatically on an automatic analyzer (Dade Behring, USA) in the Department of Clinical Laboratory at the Fangchenggang First People’s Hospital. Diabetes was defined as having a fasting blood glucose of 7.0 mmol/L or higher and/or the use of antidiabetes medication [21]. The presence of dyslipidemia was defined by serum levels of total cholesterol>6.22 mmol/L and/or LDL>4.14 mmol/L and/or triglycerides>2.26 mmol/L and/or HDL<1.04 mmol/L when compared to the normal range of Chinese adult men [22], and/or the use of cholesterol-lowering medication.

### Statistical Analysis

Initially, key demographic variables and other variables between cases (NIH-IV prostatitis) and controls (no NIH-IV prostatitis) were examined using student’s *t*-test and *chi-*squared where appropriate. The risks of NIH-IV prostatitis due to smoking and other lifestyle and medical factors were estimated by binary logistic regression models. The terms included in the model are indicated in the footnotes of the tables. We reported odds ratios (OR) with 95% confidence intervals (CI). Data management and statistical were performed with SPSS or Windows 17.0 (SPSS, Inc., Chicago, IL). Statistical tests were two-tailed, and a *P* value<0.05 was considered statistically significant.

## Results

A total of 5,988 individuals were recruited to the FAMHES during the second phase. In the end, a total of 1,868 people were included in the current cross-sectional study according to the exclusion criteria. Prevalence of NIH-IV prostatitis was 21.1% (19–29 years, 30–39 years, 40–49 years, 50–59 years, ≧60 years was 17.2%, 19.4%, 23.8%, 21.4%, 26.3% respectively) among 1,868 asymptomatic men aged 19–78 years, and 211 (11.3%) of them reported 1+, 112 (6%)/2+, 58 (3.1%)/3+, and 13 (0.7%)/4+ respectively according to leukocyte count results.

Characteristics of the study population are shown in Table 1. Men in the prostatitis group were significantly older than men in the non-prostatitis group (*P* = 0.005). The proportion of men who were smokers or drinkers showed differences between the prostatitis group and the non-prostatitis group (*P* = 0.008, *P* = 0.03). Men with NIH-IV prostatitis more likely to smoke cigarettes (≧15 Pack-years) and to drink beer, wine or hard liquor (≧ 1 drinks/week), but less likely to complete a college education (*P = *0.08). Mean levels of BMI, blood pressure, serum lipids and fasting plasma glucose were not statistically significantly different between the two prostatitis statuses. The proportion of men with physical activity, obesity, hypertension, dyslipidemia and diabetes did not show any significant difference between the prostatitis group and the non-prostatitis group.

**Table 1:** Distribution of study subjects according to presence of NIH-IV Prostatitis and selected factors.

Variables*	No Prostatitis	Prostatitis	*P*-value
	n = 1474	n = 394	
Age (yrs)	40.52 (0.30)	42.36 (0.58)	0.005
Cigarette smoking (%)			0.008
Smokers<15 Pack-years	23.70	22.10	
Smokers≧15 Pack-years	23.40	31.00	
Alcohol drinking (%)			0.03
drinkers<1 drinks/week	21.90	18.30	
drinkers ≧ 1 drinks/week	48.70	56.10	
Education of college or above (%)	57.50	52.50	0.08
Physical activity (%)	49.30	48.20	0.71
Body mass index(kg/m^2^)	24.01 (0.08)	23.90 (0.16)	0.57
Obesity (%)	11.70	10.90	0.65
Systolic blood pressure (mmHg)	123.41 (0.40)	123.72 (0.77)	0.72
Diastolic blood pressure (mmHg)	80.50 (0.25)	80.56 (0.49)	0.90
Hypertension (%)	22.00	23.10	0.68
Serum total cholesterol (mmol/L)	5.46 (0.02)	5.51 (0.05)	0.32
Serum high density lipoprotein cholestero (mmol/L)	1.29 (0.007)	1.30 (0.14)	0.79
Serum low density lipoprotein cholesterol (mmol/L)	2.82 (0.02)	2.82 (0.04)	0.97
Serum triglycerides (mmol/L)	1.92 (0.04)	1.92 (0.10)	0.99
Dyslipidemia (%)	43.50	42.60	0.77
Plasma glucose(mmol/L)	4.97 (0.03)	5.02 (0.07)	0.49
Diabetes (%)	4.20	4.80	0.57

* Mean (standard error) or percentage.

Table 2 presented the unadjusted and multivariate adjusted association between NIH-IV prostatitis and some selected factors. In the unadjusted model using binary logistic regression analysis, age≧40 years (OR = 1.35; 95% CI = 1.08–1.69; *P* = 0.008;), smokers≧15 pack-years(OR = 1.49; 95% CI = 1.14–1.93; *P* = 0.003;), alcohol consumption≧1 drinks/week (OR = 1.32; 95% CI = 1.01–1.71; *P* = 0.03) was a significant risk factor for NIH-IV prostatitis. In addition, less than a college education may was a risk factor for NIH-IV prostatitis, although statistically significant difference not existed in our data (OR = 1.22; 95% CI = 0.97–1.52; *P* = 0.08). Physical activity, overweight, hypertension, dyslipidemia and diabetes were not statistically significantly associated with NIH-IV prostatitis. Adjusted for potential confounding variables in the table (age, smoking habit, alcohol drinking habit, education, physical activity, hypertension, dyslipidemia, obesity and diabetes), age remained a significant factor for NIH-IV prostatitis (OR = 1.35; 95% CI = 1.06–1.71; *P* = 0.01) and the risk of NIH-IV prostatitis was significantly higher in smokers≧15 pack-years than non-smokers (OR = 1.33; 95% CI = 1.01–1.75; *P* = 0.03). In addition, compared with non-drinkers, the OR of NIH-IV prostatitis in drinkers ≧1 drinks/week was 1.35 (95% CI = 1.03, 1.77, *p* = 0.02) after adjusting for other variables.

**Table 2:** Adjusted odds ratios (OR) of NIH-IV Prostatitis by selected factors.

Variable	ALL	Unadjusted OR		Multivariate-adjusted OR ▽	
	(n = 1868)	OR (95% CI)	*P*-value	OR (95% CI)	*P*-value
Age (yrs)					
<40	898	1		1	
≧40	970	1.35 (1.08, 1.69)	0.008	1.35 (1.06, 1.71)	0.01
Cigarette smoking					
Never smokers	965	1		1	
Smokers<15 Pack-years	436	1.05 (0.79, 1.39)	0.67	1.07 (0.80, 1.44)	0.62
Smokers≧15 Pack-years	467	1.49 (1.14, 1.93)	0.003	1.33 (1.01, 1.75)	0.03
Alcohol drinking					
Never drinkers	534	1		1	
drinkers<1 drinks/week	395	0.95 (0.68, 1.33)	0.79	1.01 (0.72, 1.42)	0.94
drinkers≧1 drinks/week	939	1.32 (1.01, 1.71)	0.03	1.35 (1.03, 1.77)	0.02
Education of college or above					
Yes	1054	1		1	
No	814	1.22 (0.97, 1.52)	0.08	1.19 (0.94, 1.50)	0.14
Physical activity					
Yes	916	1		1	
No	952	1.04 (0.83, 1.30)	0.71	0.99 (0.79, 1.25)	0.97
Obesity					
No	1652	1		1	
Yes	216	0.92 (0.64, 1.31)	0.65	1.11 (0.77, 1.61)	0.55
Hypertension					
No	1452	1		1	
Yes	416	1.06 (0.81, 1.38)	0.65	0.98 (0.74, 1.30)	0.89
Dyslipidemia					
No	809	1		1	
Yes	1059	0.96 (0.77, 1.20)	0.76	0.91 (0.72, 1.76)	0.47
Diabetes					
No	1787	1		1	
Yes	81	1.15 (0.68, 1.95)	0.59	1.02 (0.59, 1.76)	0.91

▽ Adjusted for other variables in the table.

## Discussion

In this large cross-sectional study, the overall prevalence of NIH-IV prostatitis was 21.1% among 1,868 asymptomatic men aged 19–78 years in Fangchenggang. In previous studies, the prevalence of (symptomatic) CP was reported to be between 2% to 10% among unselected men in North America, Europe and Asia [23], and in a population based sample of Chinese men 571(4.5%) were diagnosed with prostatitis [7]. Because NIH-IV prostatitis cannot be determined by routine epidemiologic methods (questionnaires), there are only a few published studies that have reported on the prevalence of NIH-IV prostatitis. Our prevalence is similar to an earlier study of 565 young men aged 18.9±1.8 years (mean ± standard deviation), and the prevalence of NIH-IV prostatitis was 19.0% by detection of leukocytes in semen [23]. In other studies, prevalence of asymptomatic inﬂammatory prostatitis ranged from 11% to 42% [11], [24], [25]. Due to methodological differences (including detection signs of asymptomatic inﬂammation, study design and age range of the subjects) between studies, it is difficult to make a direct comparison between studies.

Although studies of the epidemiology and determinants of (symptomatic) prostatitis risk factors have provided clues to the general aetiology of prostatitis [23], information on the relationship between NIH-IV prostatitis and risk factors is lacking in the literature. Our results reveal that age is a major risk factor for NIH-IV prostatitis in Chinese men. While it has been suggested that prostatitis is a younger man’s disease, our findings are consistent with several other reports which demonstrate that prostatitis affects men of all ages [23], [26]. We also found that smokers were associated with NIH-IV prostatitis. Smoker who was smoked more than 15 pack-years have a higher risk of NIH-IV prostatitis. Meanwhile, drinkers ≧1 drinks/week also have a higher risk of NIH-IV prostatitis. Those effects remained significant after adjusting for potential confounding variables. Low education levels may be another risk factor for NIH-IV prostatitis, which can be explain by the fact that persons with lower education levels commonly pay less attention to healthcare and engage in unhealthy lifestyle practices such as smoking and drinking. However, our study did not find a significant association between physical activity, being overweight, hypertension, dyslipidemia or diabetes and NIH-IV prostatitis.

Our results reveal that men with asymptomatic inﬂammatory prostatitis may make up a significant part of asymptomatic men. Chronic prostatic inﬂammation may be involved in the development and progression of chronic prostatic disease, such as BPH(Benign Prostatic Hyperplasia) and Pca(Prostate Cancer), although there is still no evidence of a causal relation [27]. In addition, NIH-IV prostatitis has also been associated with elevated PSA levels [8], [12], and it may also be a risk factor for subsequent male infertility [28]. Asymptomatic inﬂammatory prostatitis may not be attract as much attention relative to symptomatic prostatitis, but it may be a greater hazard for people. Therefore, the prevalence of NIH-IV prostatitis should be taken into account when estimating the total prevalence of CP in future studies. Moreover, our studies have shown that unhealthy habits such as smoking and drinking increases the chances of men to suffer from prostatitis, even though they may not exhibit any symptoms. Therefore, a healthy lifestyle may be more conducive to the prevention of prostate disease.

Our study was the first large-scale epidemiological survey to investigate the prevalence of and risk factors for asymptomatic inﬂammatory (NIH-IV) prostatitis in Asian populations. Therefore, it must be informative and important for prostatitis research in the future. In our study, the population was men who voluntarily presented themselves to the physician, so their individual information about NIH-CPSI scores, lifestyle and a history of diseases should be truthful. Additionally, we excluded factors which may directly influence the measurement of NIH-IV prostatitis and we took many potential confounding variables into consideration in the multivariate logistic regression model.

Although the strength of the present study was characterized by the large sample size of the cohort population with a wide age range and multiple variables to examine, some important limitations must be recognized. First of all, we cannot test the common sexually transmitted diseases (STDs) (Neisseria gonorrhoeae, Chlamydia trachomatis, Mycoplasma genitalium and Trichomonas vaginalis) without permission, but STDs can be (both symptomatic and asymptomatic) significant reason for seminal tract inflammation. However, participants were excluded if they self-reported having specific inﬂammation (e.g. a history of s STDs) or nonspecific inﬂammation (e.g. urinary tract infections), or if they had any complaints of chronic pelvic pain or discomfort, abnormal mid void urine, or urinary tract infections. Secondly, participants with prostate diseases were more likely to get an accurate EPS collection, while self-reported co-morbidities and lifestyle might produce recall bias. Since the interview was carried out by specially trained physicians, the bias could be controlled on a small scale. In addition, the cross-sectional nature of the study has a major limitation in establishing the time sequence of events. Therefore, a follow-up study is needed to explore NIH-IV prostatitis in the future.

### Conclusions

Our findings suggest that NIH-IV prostatitis is prevalent in China. Age, smoking, drinking and lower education levels were associated with an increased risk of NIH-IV prostatitis. In additional, asymptomatic inﬂammatory prostatitis may not attract as much attention relative to symptomatic prostatitis, but may be a greater hazard for people (e.g. BPH, Pca and male infertility). Therefore, the prevalence of NIH-IV prostatitis should be taken into account when estimating the total prevalence of CP in future studies.
